# Harnessing Liposome Interactions With the Immune System for the Next Breakthrough in Cancer Drug Delivery

**DOI:** 10.3389/fphar.2019.00220

**Published:** 2019-03-12

**Authors:** Ninh M. La-Beck, Xinli Liu, Laurence M. Wood

**Affiliations:** ^1^Department of Immunotherapeutics and Biotechnology, School of Pharmacy, Texas Tech University Health Sciences Center, Abilene, TX, United States; ^2^Department of Pharmacy Practice, School of Pharmacy, Texas Tech University Health Sciences Center, Abilene, TX, United States; ^3^Department of Pharmacological and Pharmaceutical Sciences, University of Houston College of Pharmacy, Houston, TX, United States

**Keywords:** nanoparticles, tumor immunology, cancer nanomedicines, drug carrier, immunosuppression, liposome

## Abstract

Liposomal nanoparticles are a heterogeneous group of engineered drug carriers that have tremendous therapeutic potential in the treatment of cancer. They increase tumor drug delivery, significantly attenuate drug toxicity, and protect the drug from degradation. However, two decades after approval of the first nanoparticle-mediated anticancer drug, pegylated liposomal doxorubicin (Doxil), there has yet to be a major shift in cancer treatment paradigms. Only two anticancer nanoparticles are used in the first-line treatment of cancer patients, with all others relegated to the refractory or salvage setting. Herein, we discuss new insights into the mechanisms underlying *in vivo* interactions between liposomes and the tumor immunologic milieu, and the knowledge gaps that need to be addressed in order to realize the full clinical potential of cancer nanomedicines. We also discuss immunopharmacology insights from a parallel field, Cancer Immunotherapy, which have the potential to generate breakthroughs in Cancer Nanomedicine.

## Expectations for Cancer Nanomedicines

**FIGURE 1:**
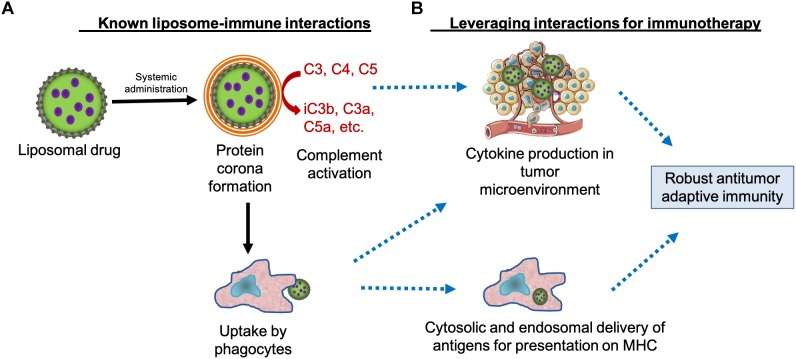
Leveraging liposome interactions with the immune system for cancer immunotherapy. **(A)** Systemically administered liposomes are known to interact with circulating proteins and cells, including components of the immune system such as immunoglobulins, complement proteins, and phagocytes. These interactions contribute to immunotoxicity and liposome clearance. **(B)** Theoretically, liposome interactions with the immune system can also be leveraged for cancer immunotherapy by stimulating cytokine production in the tumor microenvironment and by delivering tumor antigens to the requisite subcellular compartments of antigen-presenting cells, potentially generating a robust antitumor immune response. MHC, major histocompatibility complex.

Nanoparticles are a heterogeneous group of engineered drug carriers typically between 10 and 200 nm in size that include liposomes, polymers, and dendrimers. They have tremendous therapeutic potential in treatment of cancer because they increase tumor drug delivery via the enhanced permeability and retention (EPR) effect (Maeda et al., 2003), significantly attenuate drug toxicity, and protect the drug from degradation (Allen and Cullis, 2013). Liposomes are the most common nanoparticles among the approved agents, others include albumin nanoparticles and polyethylene glycol (PEG) conjugates (Anchordoquy et al., 2017). However, two decades after approval of the first nanoparticle-mediated anticancer drug, pegylated liposomal doxorubicin (PLD; Doxil), there has yet to be a major shift in cancer treatment paradigms, contrary to what was expected based on preclinical data (Petersen et al., 2016). Only two anticancer nanoparticles are used as front-line therapies: nanoparticle albumin-bound paclitaxel (*nab*-paclitaxel; Abraxane) is approved for first-line treatment of advanced non-small cell lung cancer and metastatic pancreatic adenocarcinoma, and liposomal daunorubicin cytarabine (CPX-351; Vyxeos), the only dual drug nanoparticle on the market, is approved for newly diagnosed treatment-related acute myeloid leukemia and acute myeloid leukemia with myelodysplastic changes. The reasons for suboptimal clinical efficacy of some liposomal anticancer drugs are unknown, however they are likely to involve the immune system. Liposomal nanoparticles are similar in size to pathogens such as viruses and trigger responses from the innate immune system that can lead to an increase or decrease in liposomal drug cytotoxicity, immunotoxicity, and systemic clearance (Figure 1). Herein, we focus on new insights into the mechanisms underlying *in vivo* interactions between liposomes and the tumor immunologic milieu and the knowledge gaps that need to be addressed in order to realize the full clinical potential of cancer nanomedicines. We also discuss immunopharmacology insights from a parallel field, Cancer Immunotherapy, that have the potential to generate breakthroughs in Cancer Nanomedicine.

## Interactions With Circulating Proteins

Circulating proteins rapidly adsorb to the surface of liposomes, forming a protein corona that is the interface for biological interactions (Caracciolo, 2015; Corbo et al., 2016). The mechanisms of protein adsorption and the impact of the protein corona composition on interactions with the innate immune system have been reviewed in depth (Caracciolo, 2015; Barbero et al., 2017). The protein corona contributes to particle opsonization and phagocytic clearance, and may also lead to formation of immune complexes, immunogenic epitope generation from self-antigens, and activation or suppression of immune responses (Caracciolo, 2015; Corbo et al., 2016; Barbero et al., 2017). Moreover, the protein corona can interfere with targeting functions of liposomes surface-conjugated to active targeting molecules such as antibodies (Nellis et al., 2005; Suzuki et al., 2008). Recent work in understanding the protein corona has shown that its composition is dynamic and highly variable, depending on the physicochemical characteristics of the nanoparticle as well as fluctuations in host circulating proteins. This may especially be relevant for cancer nanomedicines due to profound and heterogenous immune dysfunction associated with different types of cancer (Rosenberg, 2001). A major implication of this is that *in vitro* studies and studies in “healthy” animals are not sufficient to fully characterize the protein corona and biological impact of liposomal drugs intended for treatment of cancer.

Liposome interactions with circulating complement proteins can also lead to activation of the complement cascade (Alving, 1992; Verma et al., 1992; Szebeni et al., 2002; Dobrovolskaia et al., 2008), generating complement cleavage products that are opsonins (e.g., C3b) and fragments that are anaphylatoxins (e.g., C5a). The latter have been associated with development of acute infusion reactions in patients known as complement activation-related pseudoallergy (CARPA) (Chanan-Khan et al., 2003). Intriguingly, polymer nanoparticles that activate the complement system were found to promote tumor growth through C5a receptors (Moghimi, 2014), which increase recruitment of myeloid-derived suppressor cells (MDSCs) to the tumor microenvironment (Markiewski et al., 2008). The relevance of these findings to liposomal drugs warrants investigation since liposomes can also activate the complement cascade and generate C5a among other anaphylatoxins. Furthermore, while liposomes and other nanoparticles activate circulating complement proteins, the extent to which this occurs within tumor tissue has not been fully ascertained.

## Interactions With the Mononuclear Phagocyte System

The primary cells that interact with systemically administered liposomes are those of the mononuclear phagocyte system (MPS) such as hepatic Kupffer cells, circulating monocytes, and tissue macrophages. These interactions result in clearance of liposomal drugs from circulation and sequestration in organs of the MPS that include the liver and spleen (Caron et al., 2013; La-Beck and Gabizon, 2017). In patients, peripheral blood monocyte count (La-Beck et al., 2012) and phagocytic function (Caron et al., 2013) correlated with liposome clearance rates suggesting that functional probes of the MPS may be useful tools for personalizing treatment with nanomedicines. The incorporation of PEG polymers on liposome surfaces can reduce the non-specific adsorption of proteins and delay recognition and engulfment by the MPS (Papahadjopoulos et al., 1991; Gref et al., 2000). While phagocytic clearance of liposomes is often viewed as unfavorable in terms of drug pharmacokinetics, it has also been successfully exploited as a strategy for delivering iron-based nanoparticles to lymph nodes for imaging of occult metastases in the sentinel (tumor-draining) lymph nodes of prostate cancer patients (Fortuin et al., 2018). This supports that nanoparticles may be useful for delivery of therapies to cells and organs of the MPS. Successful examples include liposomal delivery of clodronate for depletion of tumor associated macrophages (TAMs) (Zeisberger et al., 2006), and liposomal delivery of cytotoxic chemotherapies for treatment of hepatic metastases (Gabizon et al., 1983).

## Interactions With the Tumor Microenvironment

The tumor microenvironment is composed of host derived microvasculature, stromal, and immune cells that interact with cancer cells. Certain features of the tumor microenvironment, such as hypoxia, acidity, dense extracellular matrix, can significantly impact the delivery and penetration of liposome therapeutics (Song et al., 2014). Furthermore, the tumor microenvironment is often profoundly immunosuppressed and infiltrated by cells such as regulatory T cells, TAMs, and MDSCs, that inhibit antitumor immune responses (Fearon, 1997; Rosenberg, 2001). These suppressive cells can also diminish efficacy of anticancer drugs, particularly immunotherapies (Beatty and Gladney, 2015). The impact of the tumor immunologic milieu on anticancer efficacy of cancer nanomedicines is less clear. There is increasing evidence that nanoparticles can functionally polarize macrophages (Miao et al., 2017), and that immune polarization may affect nanoparticle clearance (Jones et al., 2013). However, further work is needed to identify the precise molecular mechanisms, generalizability between different nanoparticle formulations, and downstream consequence of this on tumor progression. While classically activated (M1-like) TAMs are important in recognizing and eradicating tumor cells in the early stages, alternatively activated (M2-like) TAMs have been found to promote tumor growth and metastasis (Mantovani et al., 2002; Gabrilovich and Nagaraj, 2009). We found that in the TC-1 tumor model, *in vivo* treatment with liposomes increased expression of arginase-1 (typical of M2 macrophages) resulting in increased accumulation of TAMs with a mixed M1/M2 phenotype whereas TAMs from vehicle treated mice were predominantly M1 (Rajan et al., 2018). Moreover, others have shown that uptake of liposomes by cultured macrophages increased production of TGF-beta (Otsuka et al., 2004), consistent with the cytokine profile of immunosuppressive M2 TAMs (Allavena et al., 2008). Together, these data suggest that carrier-induced immunosuppression may partially explain why there have been an insufficient improvement in efficacy of anticancer nanoparticles in patients (Markman et al., 2004; O’Brien et al., 2004; Lammers et al., 2012; Gibson et al., 2013; Petersen et al., 2016). The underlying molecular mechanisms warrant further investigation and new insights in this area may yield breakthroughs in the application of liposomes for delivery of immunotherapies.

## Impact of Liposome-Induced Immunomodulation on Tumor Growth

**FIGURE 2:**
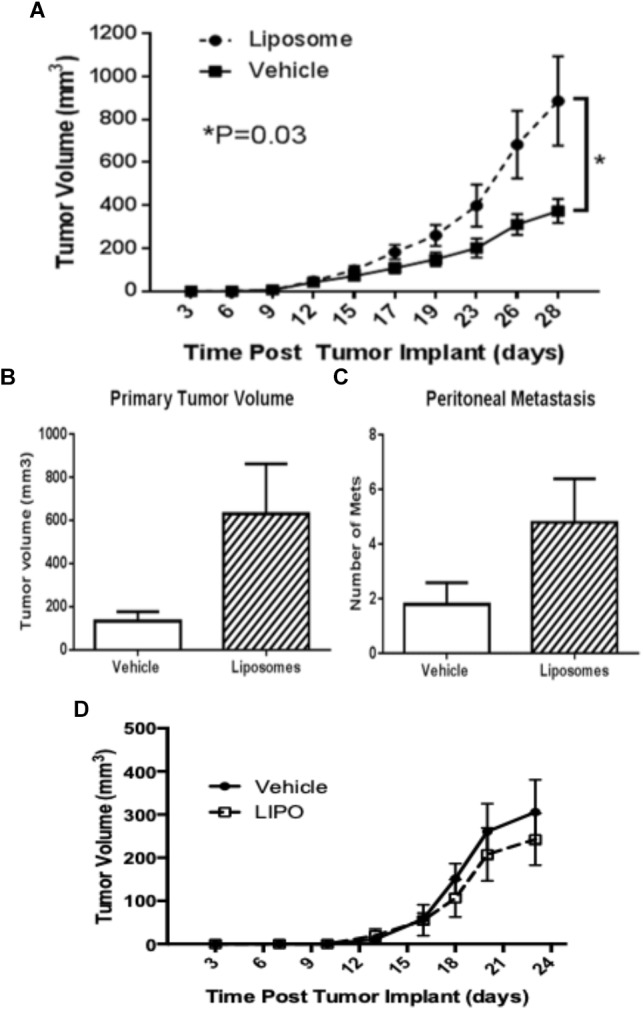
Variable impact of liposomes on tumor progression. Liposomes promote tumor growth in **(A)** TC-1 and **(B,C)** ID8-VEGF-GFP tumor models but not in **(D)** B16-OVA. Immunocompetent C57Bl/6 mice bearing **(A)** subcutaneously implanted TC-1 tumor (*n* = 16), **(B,C)** orthotopically implanted ID8-VEGF-GFP ovarian carcinoma (*n* = 10), or **(D)** subcutaneously implanted B16-OVA melanoma (*n* = 12) were treated intravenously with two weekly doses of placebo liposomes at 85 nmoles/g body weight or equivalent volume of vehicle. **(B,C)** Animals were sacrificed on Day 36. All data are mean with SEM, unpaired *T*-test.

Although liposomes interact extensively with the immune system, and the immune system is a key player in both tumor progression and regression, the impact of liposome-induced immunomodulation on tumor growth has not been systematically studied. Our initial investigations revealed that a liposomal drug carrier, similar to that used for PLD, significantly enhanced tumor growth in immunocompetent C57BL/6 mice bearing subcutaneously implanted TC-1 tumors (Figure 2A), a mouse model of HPV-induced cancer (Sabnani et al., 2015). This was associated with diminished IFN-γ production in both TAMs and cytotoxic T cells (CTLs), and decreased numbers of tumor antigen-specific CTLs in tumors compared to vehicle control (Sabnani et al., 2015). Therefore, our findings suggest that the protumoral effects of liposomes in this model are mediated by mechanisms that inhibit antitumor immunity. Importantly, we found that liposomes also increased primary tumor growth and peritoneal metastasis in C57BL/6 mice bearing orthotopically implanted ID8-VEGF-GFP tumorigenic cells, a model of ovarian cancer (Figure 2B,C), but not in C57BL/6 mice bearing implanted B16-OVA melanoma (Figure 2D). Together, these results suggest that the protumoral effects of liposomes are dependent on tumor characteristics and not on the C57BL/6 background. Further investigations are warranted to determine the extent to which these findings are generalizable to other tumor models and to cancer patients, and to identify the relationship between the physicochemical parameters of liposomes and their immune-modulatory effects. These insights could have a major impact on the clinical development of liposomal drugs for the treatment of cancer.

## Contribution of Polyethylene Glycol to Immunomodulation

Polyethylene glycol, a polymer commonly used in liposomal formulations, has been shown to illicit both immunogenic and immunosuppressive responses. While PEG can induce production of anti-PEG antibodies and activation of complement proteins, it can also induce antigen tolerance and inhibit complement activation (Schellekens et al., 2013; Verhoef and Anchordoquy, 2013). The mechanisms of PEG immunogenicity (i.e., induction of anti-PEG antibodies) are well established in preclinical models and has been associated with accelerated blood clearance of subsequent liposome treatments in animals (Ichihara et al., 2010). However, the clinical relevance of this is unclear since the opposite effect, decreased clearance of subsequent doses of liposomes, was seen in cancer patients (Gabizon et al., 2008). Moreover, while experimental animals are PEG-naïve at baseline, most cancer patients will likely have pre-existing anti-PEG antibodies due to the prevalent use of PEG in cosmetics and hygiene products (Yang et al., 2016). In contrast to these immunogenic effects, the conjugation of antigens to PEG has been shown to suppress antibody responses against the conjugated antigen (Sehon, 1991) and this approach has been utilized to optimize pharmacokinetics of various approved protein therapeutics (e.g., PEG-asparaginase) (Fu and Sakamoto, 2007). In carrier-mediated drug delivery, PEG coating (i.e., PEGylation) of the carrier particles is believed to diminish complement activation responses and evade clearance by the immune system, thereby enabling long circulating carriers (Gref et al., 2000). PEG may have additional immunosuppressive effects, as demonstrated in organ transplantation where the addition of PEG to preservation solutions has been shown to significantly improve organ function and decrease inflammation and fibrosis through suppression of the host immune responses against the transplanted organ (Tokunaga et al., 1992; Hauet and Eugene, 2008). Based on these known immunosuppressive effects of PEG, it is possible that PEG components of nanoparticles may contribute to carrier-induced suppression of antitumor immunity. This hypothesis is supported by a recent report that PEG-lipid nanoemulsions (mean particle size 125 nm) induced immunologic tolerance that was mediated by macrophages (Wang et al., 2014). However, this study did not include a non-PEG lipid control, and it is unclear whether these immunosuppressive effects were due to PEG, lipid, or both components. Nonetheless, these data strongly support a role for PEG in the immunopharmacology of nanoparticle delivered drugs that warrants further clarification.

## Overcoming Knowledge Gaps

While all preclinical models have their limitations, the current practices in testing anticancer drugs are prone to overlooking immunosuppressive and protumoral effects for three primary reasons. First, preclinical studies evaluating drugs in cancer typically focus on uncovering antitumor effects and not protumoral effects. Hence, the tumor growth rates of untreated mice in these models usually were very rapid making it difficult to further enhance tumor growth, whereas they were highly sensitive to the anti-proliferative effects of drugs. Second, the prevalent use of immune deficient mouse models (e.g., SCID) or “wild-type” mice with subtle immune defects (e.g., FVB/n which lack complement C5) may have contributed to the masking of immunomodulatory effects. Third, the preclinical evaluation of nanoparticle toxicity has historically relied on *in vitro* studies and short-term studies in animal models which are best suited for evaluating acute effects such as induction of blood complement activation and production of cytokines. In contrast, immunosuppressive effects, especially those affecting the adaptive immune system, tend to manifest after longer periods. To address current knowledge gaps, the preclinical development strategy of nanoparticle drugs should incorporate indolent tumor models, syngeneic tumors in immunocompetent mice, and *in vivo* assessments of immune responses to nanoparticle drugs. Moreover, nanoparticles are a heterogeneous group of drug carriers with multiple components and findings with one formulation cannot be assumed to be generalizable to another. The impact of physical (e.g., size and shape) and chemical (e.g., composition, steric and chemical stabilizers) properties on circulation time, direct cytotoxicity, tissue distribution, and cellular uptake has been well studied. However, although nanoparticles interact extensively with the immune system, there are few systematic studies of the relationship between physicochemical properties and *in vivo* immune modulatory activity (Ilinskaya and Dobrovolskaia, 2014). Understanding this relationship is another critical step necessary for the design of more efficacious drug carriers.

## Exploiting Immuno-Pharmacology to Maximize Anticancer Efficacy: Lessons From *Listeria*

Liposomal drug formulation strategies often focus on enhancing tumor-specific targeting of chemotherapeutic cargo while avoiding uptake by phagocytic cells. If the delivery of chemotherapeutic cargo is the only desired goal then elimination of phagocytic cell uptake may be rational. However, the natural properties of liposomes may be highly advantageous for cancer immunotherapy if the lessons learned from other delivery platforms, such as the bacterium *Listeria monocytogenes*, are applied. *Listeria monocytogenes* is a gram-positive bacterial pathogen that causes the primarily gastrointestinal disease, listeriosis (Fleming et al., 1985). After a primary exposure, subsequent challenges are met with a robust and protective immunologic memory response (Mackaness, 1962). The protective immune responses are due, in part, to the natural tropism of *Listeria*; it preferentially infects antigen-presenting cells (APCs) such as macrophages and dendritic cells (Gregory et al., 1997). Once inside the cell, it is either processed in the phagolysosome or escapes into the cytosol where it secretes bacterial antigens (Brunt et al., 1990). Ultimately, the infection leads to activation of CD4+ and CD8+ T cells that facilitate clearance of the primary infection and provide protection against subsequent exposures (Ladel et al., 1994). This tropism for APCs and robust stimulation of cytolytic immunity prompted the development of *Listeria* as a therapeutic vaccine vector for cancer treatment (Wood and Paterson, 2014). This similar propensity for liposomes to be internalized by APCs also garnered interest in developing liposomes for applications in immune-oncology.

## Enhancing Cytosolic Delivery of Antigens

Like *Listeria*, liposomes are selectively taken up by phagocytic APCs into intracellular vesicles (Jia et al., 2017). However, unmodified liposomes are inefficient at delivering molecules into the cytosol of APCs making them impractical vectors for applications requiring CTL-mediated immunity such as tumor immunotherapies (Mandal and Lee, 2002; Mandal et al., 2003). Continued development and insights learned from tumor immunotherapies, including *Listeria*, have brought renewed attention to liposomes as delivery platforms in tumor immunotherapy. One significant development is that incorporating immunoglobulins into the liposomal membrane enhances the efficiency of liposome endocytosis by APCs (Kawamura et al., 2006). Further, this resulted in greater activation of CTLs suggesting that it also increased delivery of antigens into the cytosol for processing and presentation on MHC Class I. Recently, several reports have demonstrated that incorporating the *Listeria* pore-forming toxin, Listeriolysin O (LLO), into liposomes resulted in greater release of cargo into the cytosol (Mandal and Lee, 2002; Mathew et al., 2003; Walls et al., 2013). The mechanism for this enhanced cytosolic delivery is likely derived from the normal function of LLO in the life cycle of *Listeria*. LLO is produced and secreted by *Listeria* within phagolysosomes. Activation of LLO by acidic pH, such as that found in lysosomes, causes LLO to oligomerize forming a pore within the phagolysosomal membrane (Podobnik et al., 2015). This leads to disruption of membrane integrity, allowing for *Listeria*, or in this case liposome-delivered antigens, to escape into the cytosol. Therefore, going forward, liposomes may employ a number of strategies concurrently, including incorporation of *Listeria*-derived LLO, to optimally deliver tumor-associated antigen cargo to the MHC Class I processing/presentation machinery and activate a robust antitumor CTL response.

## Co-Delivery of Immunostimulatory Molecules

A robust and therapeutic antitumor CTL response is advanced not only by efficient delivery of antigens, but also by stimulation of inflammatory cytokine production. Several groups have developed strategies to enhance the antitumor efficacy of liposome-based immunotherapies by incorporating pathogen-associated molecular patterns (PAMPs) and other immunostimulatory molecules (Nisini et al., 2018). One notable example that has proceeded through phase III clinical trials is Stimuvax, a liposome-based vaccine for the treatment of melanoma. Stimuvax is formulated with the lipid A portion of lipopolysaccharide to stimulate Toll-like receptor 4-mediated inflammation (Wu et al., 2011). Going forward, liposome-based vaccines incorporating PAMPs such as lipoteichoic acid or cyclic di-GMP from *Listeria monocytogenes* may improve clinical efficacy. In fact, incorporation of multiple PAMPs that target different pattern recognition receptors (PRRs) may be the most effective approach for clinical applications due to the prevalence of PRR polymorphisms in the human population (Medvedev, 2013). In addition to PAMPs as activators of innate immunity, incorporation of protein antigens for pre-existing T helper memory responses could provide an even greater benefit in the initial priming of an antitumor CTL response. As most adults are immunized against measles, mumps, tetanus, and other infectious diseases during childhood, the inclusion of antigens from these vaccines along with tumor-associated antigens could leverage the pre-existing pool of memory T helper cells to enhance antitumor responses as previously proposed for *Listeria*-based vaccines (Chandra et al., 2016).

Like liposomes, *Listeria* preferentially accumulates in tumors (Yu et al., 2004) and may be protected from elimination by the immunosuppressive tumor microenvironment (Fearon, 1997). This tumor tropism by *Listeria* has been leveraged pre-clinically to deliver pro-drug metabolizing enzymes and radioactive isotopes to tumors that result in direct cytotoxicity and, likely, enhanced anti-tumor immunity (Stritzker et al., 2008; Quispe-Tintaya et al., 2013; Medler et al., 2015). In fact, a recent study suggests that adding cytotoxic chemotherapy to immunostimulatory nanoparticles leads to enhanced anti-tumor efficacy (Chen et al., 2016). The extensive clinical experience with liposomes as drug delivery vectors suggests that they would be ideal for such an application. Further, liposomes would benefit from being able to deliver chemotherapeutic agents that may be toxic to bacterial vectors while avoiding the rare but unique challenges of live attenuated vectors (Denham et al., 2018; Fares et al., 2018; Papanicolas et al., 2018).

## Future Directions: Leveraging Liposomes for Tumor Immunotherapy

The future of liposomes and other nanoparticles in tumor immunotherapy is promising if the lessons learned from development of other vaccine vectors continue to be effectively translated. Drug delivery using liposomes has been a valuable strategy to mitigate toxicity of anticancer drugs in patients (Gibson et al., 2013; Golan et al., 2015; Gabizon et al., 2016). While this approach has not significantly improved clinical efficacy thus far (Petersen et al., 2016), new understanding of the mechanisms of interactions between liposomes and the immune system will lay the foundation for future work that will realize the full clinical potential of cancer nanomedicines (La-Beck and Gabizon, 2017).

## Data Availability

All datasets generated for this study are included in the manuscript and/or the supplementary files.

## Ethics Statement

This study was carried out in accordance with the recommendations of the Texas Tech University Health Sciences Center (TTUHSC) Institutional Animal Care and Use Committee (IACUC). The protocol was approved by the TTUHSC IACUC.

## Author Contributions

NL-B and LW contributed equally to conception and writing of this manuscript. XL contributed to writing this manuscript. All authors revised and approved the final version.

## Conflict of Interest Statement

The authors declare that the research was conducted in the absence of any commercial or financial relationships that could be construed as a potential conflict of interest.
